# Investigating Crosstalk Among PTMs Provides Novel Insight Into the Structural Basis Underlying the Differential Effects of Nt17 PTMs on Mutant Httex1 Aggregation

**DOI:** 10.3389/fmolb.2021.686086

**Published:** 2021-07-26

**Authors:** Anass Chiki, Zhidian Zhang, Kolla Rajasekhar, Luciano A. Abriata, Iman Rostami, Lucien F. Krapp, Driss Boudeffa, Matteo Dal Peraro, Hilal A. Lashuel

**Affiliations:** ^1^ Laboratory of Molecular and Chemical Biology of Neurodegeneration, School of Life Sciences, Brain Mind Institute, Ecole Polytechnique Fédérale de Lausanne (EPFL), Lausanne, Switzerland; ^2^ Laboratory for Biomolecular Modeling, School of Life Sciences, Institute of Bioengineering, Ecole Polytechnique Fédérale de Lausanne (EPFL), Lausanne, Switzerland; ^3^ Laboratory of Biomolecular Research, Department of Biology and Chemistry, Paul Scherrer Institute, Villigen, Switzerland

**Keywords:** post-translational modifications, huntingtin, molecular dynamics simulation, chemical semi-synthesis, atomic force microscope

## Abstract

Post-translational modifications (PTMs) within the first 17 amino acids (Nt17) of the Huntingtin protein (Htt) have been shown to inhibit the aggregation and attenuate the toxicity of mutant Htt proteins *in vitro* and in various models of Huntington’s disease. Here, we expand on these studies by investigating the effect of methionine eight oxidation (oxM8) and its crosstalk with lysine 6 acetylation (AcK6) or threonine 3 phosphorylation (pT3) on the aggregation of mutant Httex1 (mHttex1). We show that M8 oxidation delays but does not inhibit the aggregation and has no effect on the final morphologies of mHttex1aggregates. The presence of both oxM8 and AcK6 resulted in dramatic inhibition of Httex1 fibrillization. Circular dichroism spectroscopy and molecular dynamics simulation studies show that PTMs that lower the mHttex1 aggregation rate (oxM8, AcK6/oxM8, pT3, pT3/oxM8, and pS13) result in increased population of a short N-terminal helix (first eight residues) in Nt17 or decreased abundance of other helical forms, including long helix and short C-terminal helix. PTMs that did not alter the aggregation rate (AcK6) of mHttex1 exhibit a similar distribution of helical conformation as the unmodified peptides. These results show that the relative abundance of N- vs. C-terminal helical conformations and long helices, rather than the overall helicity of Nt17, better explains the effect of different Nt17 PTMs on mHttex1; thus, explaining the lack of correlation between the effect of PTMs on the overall helicity of Nt17 and mHttex1 aggregation *in vitro*. Taken together, our results provide novel structural insight into the differential effects of single PTMs and crosstalk between different PTMs in regulating mHttex1 aggregation.

## Introduction

Huntington’s disease (HD) is a fatal, autosomal neurodegenerative disease characterized by motor (Huntington, 1872; Ross and Tabrizi, 2011) and cognitive declines (Marder et al., 2000) as well as psychiatric symptoms (Paulsen et al., 2001). HD is caused by a mutation in the huntingtin gene (*HTT*), resulting in an expansion in the CAG repeat within its first exon (Gusella et al., 1983; MacDonald, 1993), which is then translated into an extended polyglutamine (polyQ) repeat in the huntingtin protein (Htt) (Kremer et al., 1994). HD occurs when the length of the polyQ repeat is higher than the critical threshold of ≥36 (Snell et al., 1993). At the neuropathological level, HD is characterized by neuronal degeneration in the striatum and the cortex (Reiner et al., 1988; Rosas et al., 2003) and the formation and accumulation of nuclear inclusions composed of mutant Htt (polyQ repeat ≥36) aggregates and other proteins (DiFiglia et al., 1997; Hodgson et al., 1999). Several studies have shown that these inclusions are composed of fibrillar and potentially oligomeric species derived from N-terminal fragments containing expanded polyQ repeats (Mangiarini et al., 1996; DiFiglia et al., 1997; Lunkes et al., 2002; Landles et al., 2010; Sathasivam et al., 2013). One of the major N-terminal fragments found in these inclusions represents an N-terminal fragment that corresponds to exon1 of the Htt protein (Httex1) (Landles et al., 2010; Sathasivam et al., 2013). Overexpression of mutant Httex1 alone with polyQ length ranging from 80 to 175 reproduces many aspects of HD pathology in various animal and cellular models, including the formation of huntingtin inclusions (Mangiarini et al., 1996; Martindale et al., 1998; Scherzinger et al., 1999; Barbaro et al., 2015).

**FIGURE 1:**
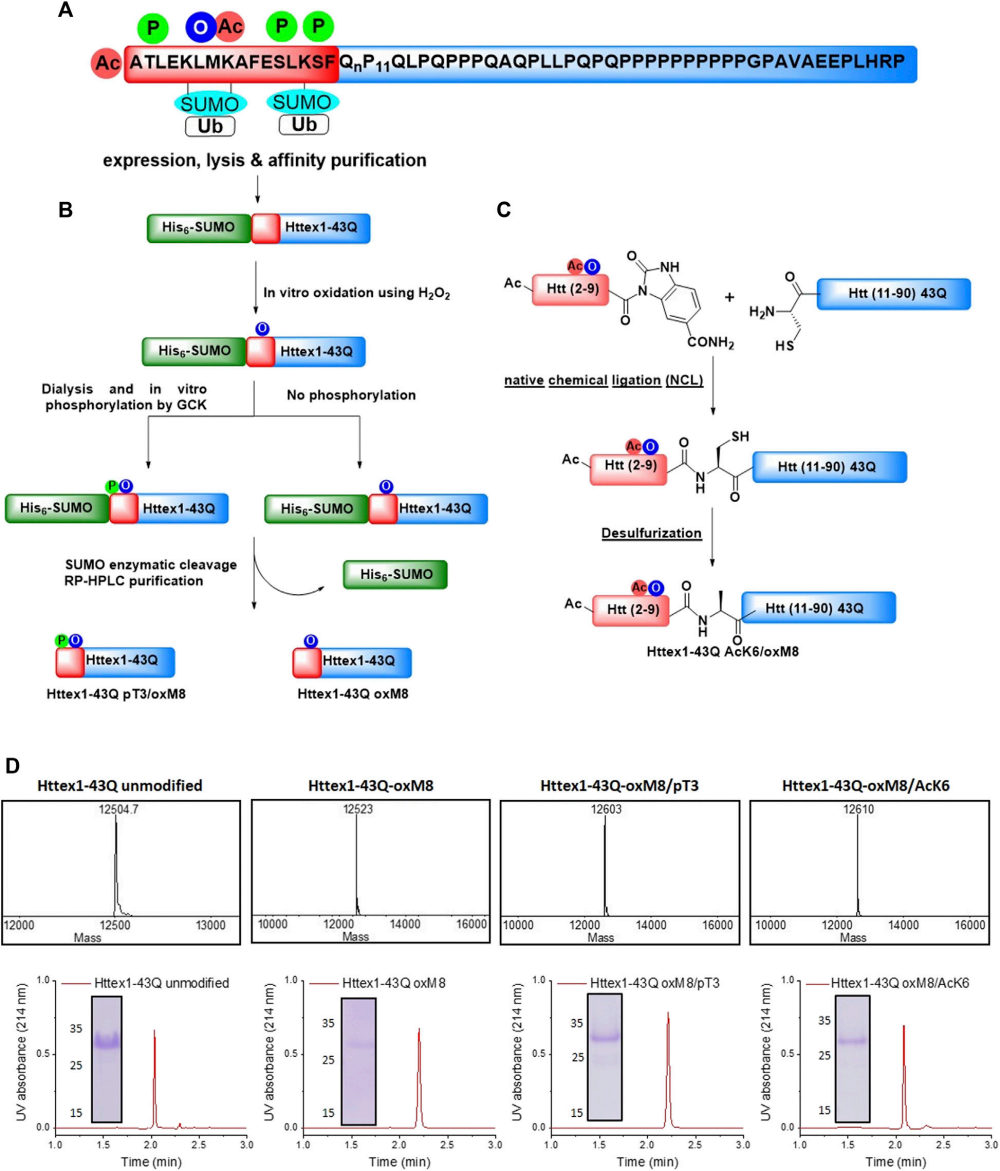
Chemical and enzymatic methods for the generation of Httex1-43Q-oxM8, Httex1-43Q-oxM8/pT3, and Httex1-43Q-oM8/AcK6. **(A)** Schematic presentation of the Httex1 sequence, highlighting the cluster of PTMs in the Nt17 domain. **(B)** Schematic presentation of the SUMO-based strategy used to produce Httex1-43Q-oxM8 and Httex1-43Q-oxM8/pT3. The SUMO-Httex1-43Q was produced and purified by Nickel IMAC purification and subsequently oxidized or both oxidized and phosphorylated by GCK. Next, the SUMO tag was removed by ULP1, and the desired protein was purified by RP-HPLC. **(C)** Schematic representation for the semisynthetic strategy used for the generation of mHttex1-oxM8/AcK6 (adapted from Chiki et al. (2017)). **(D)** Characterization of Httex1-43Q-oxM8, Httex1-43Q-oxM8/pT3, Httex1-43Q-oM8/AcK6, and unmodified Httex1-43Q by ESI/MS, UPLC, and SDS-PAGE.

Although increasing evidence suggests that mutant Htt aggregation and toxicity play central roles in HD’s pathogenesis, the molecular events responsible for triggering mutant Htt aggregation, the nature of the toxic species, and the mechanisms by which they cause neurodegeneration remain unknown. Initial efforts focused on disentangling the relationship between Htt aggregation and toxicity and HD have focused on identifying modifiers of Htt aggregation based on targeting the polyQ repeat domain, which has been shown to be the primary sequence responsible for initiating Htt aggregation. However, recent studies suggest that post-translational modifications (PTMs) in close proximity or far from the polyQ domain have the potential to modify not only mutant Htt levels and functions, but also its aggregation and toxicity (Thompson et al., 2009; Aiken et al., 2009; Steffan et al., 2004; Gauthier et al., 2004; Warby et al., 2005; Yanai et al., 2006; Jeong et al., 2009; O’Rourke et al., 2013). Interestingly, several of these PTMs occur within the first N-terminal 17 amino acids of Htt, directly flanked by the polyQ domain (Figure 1A). These modifications include phosphorylation at multiple serine and threonine residues (T3, S13, and S16), acetylation, ubiquitination, and SUMOylation at selected lysine residues (K6, K9, K15) (Figure 1A). Mutating both S13 and S16 to aspartate to mimic the phosphorylation was shown to reverse the pathology of mutant Htt in an HD mouse model (Gu et al., 2009) and modulate Htt aggregation in different cellular models (Mangiarini et al., 1996; Martindale et al., 1998; Scherzinger et al., 1999; Barbaro et al., 2015). Recently, we showed that phosphorylation at T3, S13, and/or S16 inhibited the aggregation of WT and mutant Httex1 *in vitro* (Chiki et al., 2017; DeGuire et al., 2018).

Furthermore, TBK1 (TANK-binding kinase 1) mediated phosphorylation of mutant Httex1 at S13 and S16 lowers its levels and results in a significant reduction in Httex1 aggregation and inclusion formation in different cellular models and a *C. elegans* model of HD (Hegde et al., 2020). These findings, combined with our observation that the levels of phosphorylated Htt at T3 are decreased in pathological conditions (Cariulo et al., 2017), suggest that phosphorylation within the Nt17 domain protects against mutant Htt aggregation. Similarly, SUMOylation at multiple N-terminal lysine residues inhibits Httex1 aggregation *in vitro* (Sedighi et al., 2020). Interestingly, although lysine acetylation at K6, K9, or K15 does not significantly alter the aggregation profile of Httex1 (Chiki et al., 2017), acetylation at K6 was shown to significantly inverse the inhibitory effects of phosphorylation at T3 on aggregation (Chiki et al., 2017). Similarly, phosphorylation of S13 and S16 was shown to regulate Httex1 acetylation at K9 (Thompson et al., 2009), and ubiquitination/SUMOylation at K6 and K9 (Thompson et al., 2009; O’Rourke et al., 2013). Finally, competing modifications, such as ubiquitination and SUMOylation, were shown to exert different effects on Htt levels and degradation (Steffan et al., 2004). Taken together, these findings, combined with the fact that multiple reversible PTMs cluster within eight amino acids in Nt17, suggest that many of these modifications act in concert rather than individually and that the Htt PTM code involves crosstalk between different PTMs, in particular, those that exist close to each other.

One PTM that remains unstudied and its effect on mutant Htt aggregation remains unknown is the oxidation of methionine 8 (oxM8). This residue is highly conserved (DiGiovanni et al., 2016), and the levels of M8 oxidation were shown to be higher in the R6/2 HD mouse model (Mitomi et al., 2012). However, the absence of immunochemical methods to detect M8 oxidation has precluded studies aimed at understanding its relevance and potential roles in HD neuropathology. Despite this, methionine oxidation is one of the most common modifications that occur under oxidative stress (Hoshi and Heinemann, 2001), and has been linked to the pathogenesis of HD (Kumar and Ratan, 2016) and other neurodegenerative diseases. Elevated levels of oxidative stress markers, including reactive oxygen species (ROS), were found in blood (Chen et al., 2007) and postmortem brains of HD patients (Polidori et al., 1999). Although there is no direct evidence establishing that M8 is oxidized, several studies have suggested that this could occur under oxidative stress conditions and that M8 oxidation could act as a sensor of ROS to regulate Htt phosphorylation and localization (DiGiovanni et al., 2016; Son et al., 2019). Mitomi and colleagues (Mitomi et al., 2012) showed, using hydrogen peroxide (H_2_O_2_) mediated oxidizing conditions *in vitro*, that oxidation at M8 occurs only post-aggregation on non-soluble forms of mutant Httex1. Recent studies based on quantitative NMR showed that TiO_2_ nanoparticles induced M8 oxidation and abolished the aggregation of a model peptide of Httex1, and reduced the model peptide binding to lipids micelles (Ceccon et al., 2018). However, most of these studies were carried out either using Nt17 peptides, GST-tagged Httex1 protein (Mitomi et al., 2012), or a model peptide consisting of only the Nt17 linked to short polyQ tracts of only 7 or 10 glutamines, lacking the proline-rich domain and most the C-terminal domain of Httex1 (Ceccon et al., 2018). Additionally, in some studies, the H_2_O_2_ was not removed from the solution prior to the aggregation experiments.

Here, we used an integrative approach combining biophysical and computational studies to gain insight into the effect of M8 oxidation and potential crosstalk between M8 oxidation with neighboring PTMs in regulating the structure and aggregation of mutant Httex1. Toward this goal, we used chemoenzymatic approaches to produce site-specific modified Httex1 proteins bearing different PTMs, including: 1) oxM8, 2) oxM8 and phosphorylation at T3, and 3) oxM8 and acetylation at K6. With these proteins in hand, we performed systematic studies to understand the effect of M8 oxidation and its crosstalk with pT3 and AcK6 in modulating the structure, aggregation, and fibrillar morphology of Httex1. To further understand the structural basis underlying the effects of these modifications on mutant Httex1 aggregation, we performed circular dichroism (CD) analysis and atomistic molecular simulations to investigate how these different combinations of PTMs influence Nt17 structure and helicity. Our findings provide new insight into the PTM code of Nt17, and the differential effects of PTM-induced conformational changes on mutant Httex1 (mHttex1) aggregation.

## Results

### Production of Oxidized Mutant Httex1 Proteins

Httex1 contains two methionine residues within its Nt17 domain. The N-terminal methionine is cleaved by aminopeptidase resulting in N-terminally acetylated alanine as the first residue (Arnesen et al., 2010). This means that once expressed, Httex1 contains only a single methionine residue at position 8 (M8). Investigating the biological role of M8 oxidation is more challenging than other Nt17 PTMs, such as phosphorylation, because there are no natural amino acids that mimic methionine oxidation and factors that are known to induce protein oxidation, such as oxidative stress, are not always chemoselective. Therefore, to investigate the effect of M8 oxidation on Httex1 aggregation, we sought to prepare mutant Httex1 proteins that are homogeneously oxidized at M8. Several protocols have been used to oxidize methionine residues in proteins *in vitro*, including treatment with H_2_O_2_, transition metal ions, 2,2-azobis (2-amidinopropane) dihydrochloride (AAPH), tert-butyl hydroperoxide (t-BHP), and UV exposure (Keck, 1996; Wasylaschuk et al., 2007; Ji et al., 2009; Luo et al., 2011).

In the context of Httex1, previous studies have shown that treatment with H_2_O_2_ allows for efficient oxidation of M8 within a GST-Httex1 fusion protein (Mitomi et al., 2012), Nt17 peptides (DiGiovanni et al., 2016), or Httex1 model peptides consisting of Nt17 domain with ten additional glutamine residues (Nt17Q10) (Ceccon et al., 2018). Some of the limitations of these studies are: 1) none of the proteins used represent the native sequence of Httex1; 2) the extent of oxM8 was not always confirmed by mass spectrometry or other methods; or 3) in some studies, the structural or aggregation properties of the protein and peptide models was assessed in the presence of the oxidizing agent, H_2_O_2_. It is known that incubation of proteins under such harsh conditions for an extended period might modify other residues within the sequence, such as histidine or phenylalanine (Li et al., 1995), which could alter the sequence and biophysical properties of Httex1.

To overcome these limitations, we developed a strategy for producing native mutant Httex1 specifically oxidized at M8 (oxM8). We took advantage of recent advances from our lab that allow for the generation of milligram quantities of highly pure mutant Httex1 fused to the SUMO protein (Reif et al., 2018) (Figure 1B). The desired PTMs (oxidation or phosphorylation) are then introduced, using enzymatic or chemical approaches, into Nt17 of the fusion protein, followed by removal of the SUMO protein and purification of the modified mutant Httex1 by RP-HPLC (Chiki et al., 2020).

Mutant Httex1, with 43 glutamine residues (mHttex1) fused to a SUMO tag protein at its N-terminal (SUMO-mHttex1), was expressed and purified as previously described (Supplementary Figure 1A,B) (Reif et al., 2018). Next, the fusion protein was subjected to oxidation using 400 mM of H_2_O_2_ in 50 mM Tris, 500 mM NaCl, 500 mM Imidazole, pH 7.5. The oxidation reaction was monitored over time by ESI/MS (Supplementary Figure 1C). For each time point, an analytical scale cleavage reaction of the SUMO tag using the ULP1 enzyme was performed, and the resulting cleaved product was analyzed by ESI/MS. As shown in Supplementary Figure 1C, the M8 residue was completely oxidized after 2 h of incubation with H_2_O_2_. The oxidized SUMO-mHttex1 was then subjected to ULP1 cleavage. Upon verifying the complete removal of the SUMO tag, *m*-Httex1-oxM8 was immediately purified by reverse phase HPLC (RP-HPLC) (Supplementary Figure 1E). The fractions containing the protein of interest were then pooled together and lyophilized. The final purity of the mHttex1-oxM8 was verified by ESI/MS, UPLC, and SDS-PAGE (Figures 1D).

To investigate the effect of potential crosstalk between oxM8 and other Nt17 PTMs on mutant Httex1 aggregation, we generated mutant Httex1 proteins oxidized at M8 and phosphorylated at T3 (pT3) or acetylated at K6 (AcK6). To produce mHttex1 oxidized at M8 and phosphorylated at T3 (mHttex1-oxM8/pT3), we first generated SUMO-mHttex1-oxM8 as described above. After overnight dialysis to remove the excess of H_2_O_2_, the fusion protein was co-incubated overnight with GCK kinase, a kinase that we recently reported to efficiently phosphorylate Httex1 specifically at T3 (Chiki et al., 2020). The extent of phosphorylation was monitored by ESI/MS. As shown in Supplementary Figure 1D, the ESI/MS spectrum of the SUMO-mHttex1-oxM8 showed an additional +80 Da, indicating the addition of a single phosphate group. The SUMO-mHttex1-oxM8/pT3 was then subjected to UPL1 cleavage followed by RP-HPLC purification of mHttex1-oxM8/pT3 (Supplementary Figure 1F). The protein’s purity was verified by ESI/MS, UPLC, and SDS-PAGE (Figure 1D).

To produce mutant Httex1 that is both oxidized at M8 and acetylated at lysine 6, we employed a semisynthetic protein strategy (Figure 1C) that we previously used to introduce single or multiple PTMs within Nt17 of mHttex1 (Chiki et al., 2020). mHttex1-AcK6/oxM8 was produced using native chemical ligation between the Htt A10C-90 43Q and Ac-2-9-Nbz AcK6/oxM8 peptides (characterization by ESI-MS and UPLC is shown in Supplementary Figure 2B), followed by desulfurization to convert cysteine 10 back to the native alanine (Supplementary Figure 2C). The purity of mHttex1 AcK6/oxM8 was determined by SDS-PAGE, UPLC, and ESI/MS (Figure 1D).

### Oxidation at M8 Delays the Aggregation of Mutant Httex1

**FIGURE 2:**
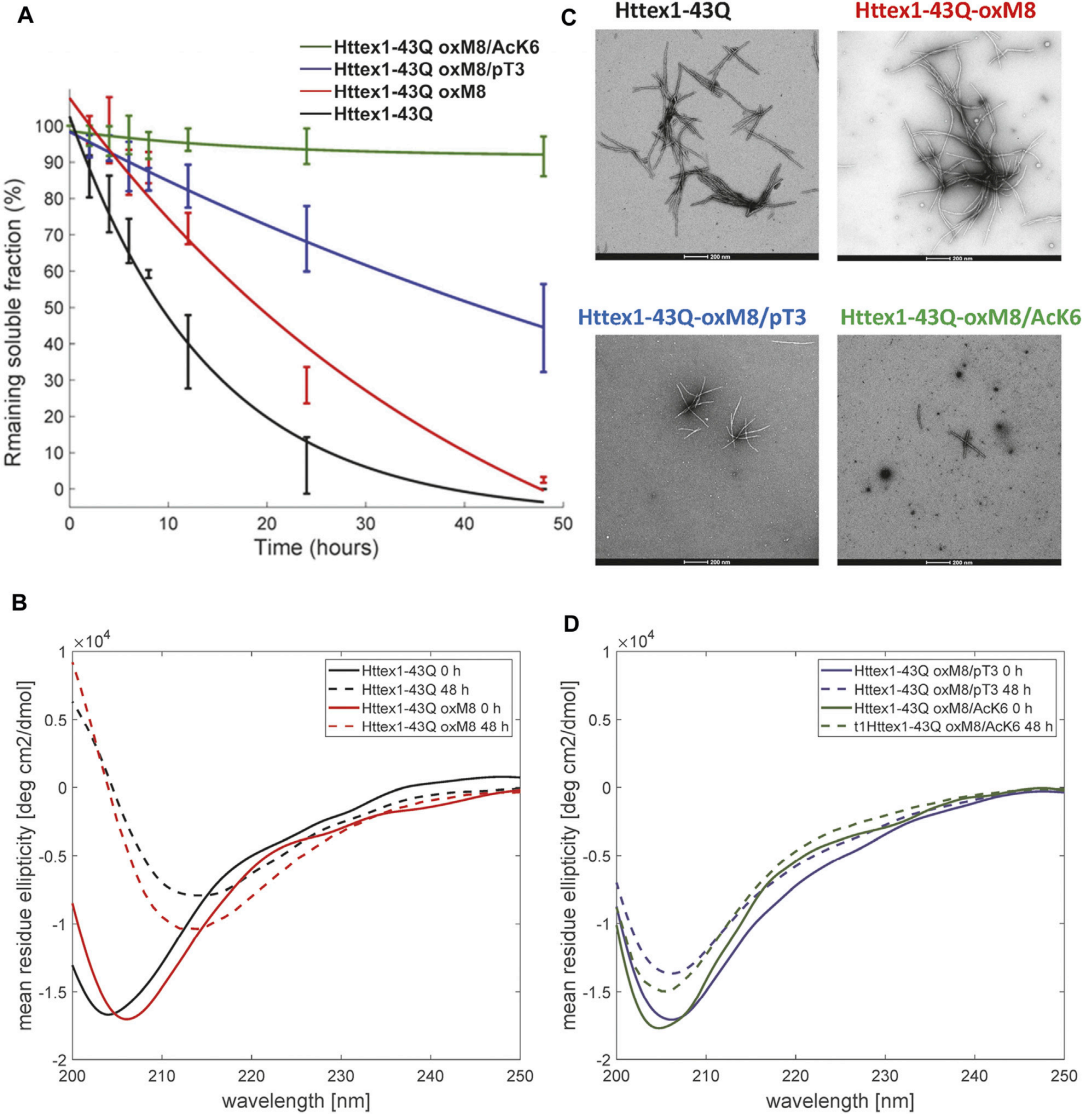
Biophysical studies to elucidate the effect of pT3, OxM8, and AcK6 on mutant Httex1 aggregation. **(A)** Httex1 aggregation studies (at 5 µM) of Httex1-43Q-oxM8, Httex1-43Q-oxM8/pT3, Httex1-43Q-oM8/AcK6, and unmodified mHttex1 monitored by UPLC sedimentation assay to determine the proportion of remaining monomer (error bars, S.D. derived from *n* = 3 independent experiments fitted with exponential decay). **(B)** Secondary structure analysis of unmodified Httex1-43Q and Httex1-43Q-oxM8 by CD at 0 and 48 h of aggregation. **(C)** Electron microscopy after 48 h of aggregation (scale bars = 200 nm). **(D)** Secondary structure analysis of Httex1-43Q-oxM8 and Httex1-43Q-oxM8 by CD at 0 and 48 h post-aggregation initiation.

To determine the effect of oxM8 on mutant Httex1 aggregation, mHttex1-oxM8, and mHttex1 were subjected to a disaggregation protocol, as previously reported (Reif et al., 2018). Any remaining aggregates were removed by filtration through a 100 kDa filter before initiating the aggregation. The extent of and kinetics of aggregation were monitored by quantifying the amount of soluble proteins at different time points using a previously described UPLC-based sedimentation assay (Vieweg et al., 2016; O'Nuallain et al., 2006a; Jayaraman et al., 2011). Also, the effect of oxidation on the secondary structure and morphology of the fibrils was also assessed by circular dichroism spectroscopy (CD) and electron microscopy (EM). Figure 2A shows the percentage of the remaining soluble protein over time. As expected, unmodified mHttex1 exhibited almost complete depletion of the soluble monomer (Figure 2A) and full conversion into aggregates after 48 h, which was confirmed by a shift in the CD spectra from random coil to a *ß*-sheet structure (Figure 2B) and the presence of mature long fibrils (Figure 2C). In contrast, mHttex1-oxM8 showed a delay in aggregation compared to the unmodified mHttex1 (Figure 2A). The aggregation of mHttex1-oxM8 showed 68 and 39% remaining monomer after 12 and 24 h, respectively, compared to 40 and 13% for the unmodified mHttex1 (Figure 2A). However, after 48 h, both proteins exhibited complete aggregation as discerned by the complete disappearance of soluble Httex1 (Figure 2A) and the CD spectra of both proteins, which showed a signal that corresponds to predominantly *ß*-sheet-rich structures (Figure 2B). The fibrils formed by mHttex1 oxM8 after 48 h were similar to those formed by the unmodified mutant Httex1, suggesting that oxM8 influences aggregation’s kinetics but not the final structure of the fibrils (Figure 2C).

**FIGURE 3:**
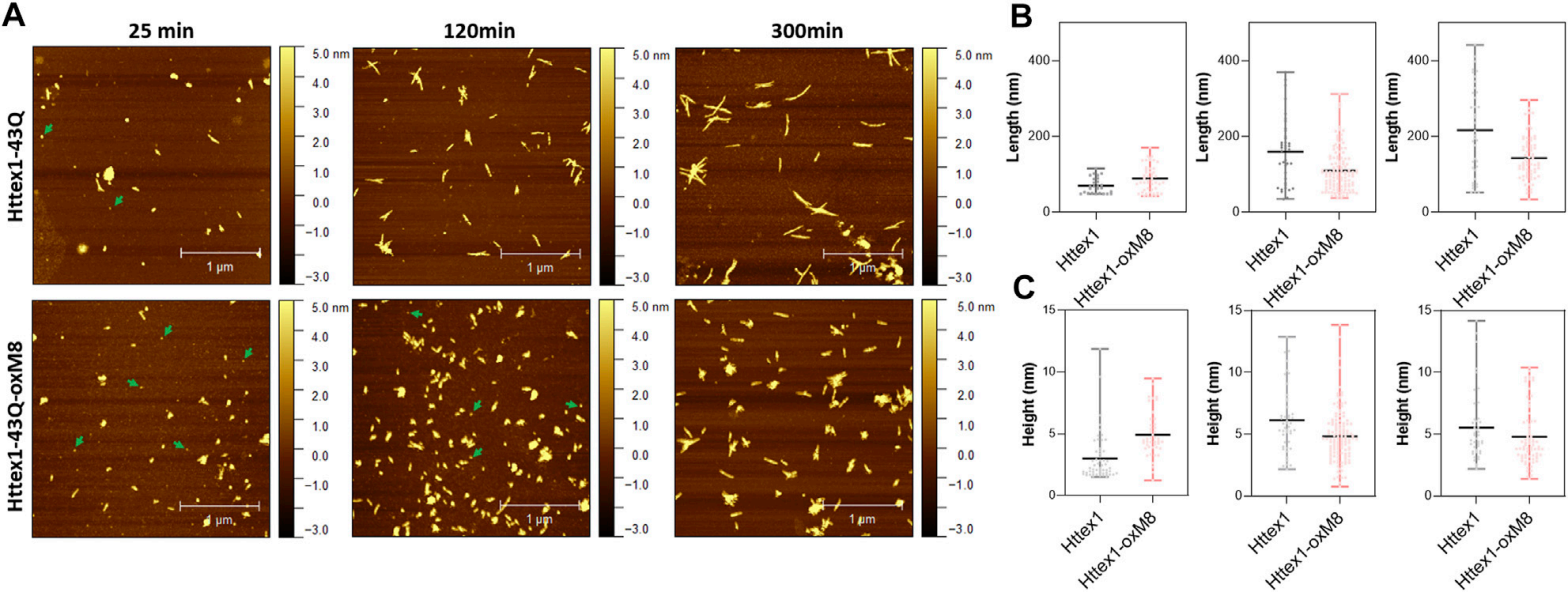
AFM analysis comparison between unmodified and Httex1-43Q-OxM8. **(A)** Time-dependent AFM images of aggregates formed by Httex1-43Q and Httex1-43Q-oxM8 (nm, nanometer), the oligomers are indicated with green arrows. Quantitative analysis of the length **(B)** and height **(C)** of fibrils measured from AFM images. All the plots for the length and height of the Httex1-43Q-oxM8 fibrils differ from the Httex1-43Q with a statistical significance of at least *p* < 0.05 (scattered plot represents the mean and range).

Interestingly, we consistently observed the presence of a population of oligomers for the oxidized Httex1 at early time points (Figure 3 and Supplementary Figure 3). These oligomer populations were observed in a higher amount after 4 h of aggregation by EM, at which unmodified mHttex1 already showed fibrils formation (Supplementary Figure 3). After 6 h, mHttex1-oxM8 showed a mixture of oligomers and short fibrils (Supplementary Figure 3), whereas only fibrils were observed in the case of the unmodified mHttex1. To further validate our observations and quantitatively assess differences in dimensions and morphological properties of the aggregates formed by the two proteins, we performed time-dependent atomic force microscopy (AFM) studies. Consistent with our EM analysis, unmodified mHttex1 and mHttex1-oxM8 showed the presence of both fibrils and oligomers at 25 min, as shown in Figure 3A. Interestingly, at 2 h, mHttex1 predominantly formed longer fibrils with a mean length of 167 nm compared to mHttex1 oxM8, which formed a mixture of oligomers and short fibrils (111 nm). Similarly, after 6 h of incubation, unmodified mHttex1 formed fibrils with a mean length of 215 nm and a height of 5.5 nm (Figure 3), whereas mHttex1 oxM8 samples showed a mixture of shorter fibrils and protofibril-like structures with average lengths and heights of 140 and 4.8 nm, respectively (Figure 3). These observations are consistent with the slower aggregation kinetics observed for the mHttex1 oxM8 in the sedimentation assay. Taken together, these results suggest that the delay in the aggregation of the oxidized mutant Httex1 could be associated with the formation and accumulation of oligomers (on- or off-pathway), that eventually convert into fibrils at later stages.

### Crosstalk Between M8 Oxidation and Phosphorylation at T3 or Acetylation at K6

To determine the effect of PTM crosstalk on aggregation within the Nt17 domain, we investigated and compared the aggregation kinetics and properties of Httex1-oxM8/pT3 and -oxM8/AcK6. Previously, we showed that >60% of mHttex1-pT3 remains soluble after 48 h of aggregation, indicating an inhibitory effect of T3 phosphorylation on mutant Httex1 *in vitro* (Chiki et al., 2017). As shown in Figure 2, the oxidation of M8 does not seem to influence the inhibitory effect of phosphorylation at T3. Even after 48 h of incubation, ∼50% of the protein remained soluble, whereas more than 98% of the unmodified proteins have been converted to fibrillar aggregates (Figure 2A). The CD spectrum showed that the remaining protein retains a random coil signature, suggesting that the remaining soluble protein represents monomers or disordered soluble oligomers (Figure 2D). Analysis by EM showed the accumulation of oligomers and short fibrils similar to those we previously reported for mHttex1-pT3 (Chiki et al., 2017). On the other hand, oxidation of M8 in the context of the K6 acetylated mutant Httex1 dramatically altered the aggregation properties of the protein. Previously, we have shown that acetylation of K6 or K9 does not significantly influence the aggregation of mutant Httex1. However, as shown in Figure 2A, the presence of both acetylation at K6 and methionine oxidation at M8 (mHttex1-oxM8/AcK6) results in significant inhibition of mutant Httex1 aggregation. Only 8% of mHttex1 oxM8/AcK6 aggregated after 48 h (Figure 2A) as determined by the sedimentation assay and confirmed by CD and EM, which show predominantly disordered conformation and the accumulation of only oligomers and small fibrils (Figure 2C,D). These results suggest that the addition of oxM8 reversed the aggregation of mHttex1-AcK6 and illustrates how the combination of different Nt17 PTMs could differentially influence the aggregation properties of the protein.

### Oxidation at M8 Decreases the Helicity of the Nt17 Peptides

To gain insight into the mechanisms by which oxM8 and its combination with other PTMs influence the aggregation of mutant Httex1, we investigated how the individual Nt17 PTMs and different combinations of PTMs influence on the structural properties of the Nt17 domain. We confirmed that Nt17-AcK6 exhibited increased helical content (Pastore et al., 2019) compared to the Nt17-WT peptide (Supplementary Figure 4) (Pastore et al., 2019). Additionally, by CD, we observed that Nt17-pT3 has a higher propensity to adopt helical conformation compared to other Nt17 peptides (Supplementary Figure 4, Table 1). When oxM8 was added to Nt17-AcK6 and Nt17-pT3, we observed a reduced CD signal at 222 nm for the Nt17-oxM8/pT3 and Nt17-oxM8/AcK6 peptides (Supplementary Figure 4), indicating a decrease in the helical content which was confirmed by determining their secondary structure content from the CD spectra (See Supplementary Table 1).

Furthermore, to investigate if the decrease in the helicity induced by oxM8 persists even at a high concentration of the peptides, we conducted CD studies (Supplementary Figure 5A) at different Nt17 peptide concentrations (60, 90, 120, 150, and 200 μM). As shown in Supplementary Figure S5, all of the peptides showed a small increase in the helical content with increasing concentrations (Supplementary Figure 5B). oxM8 slightly decreased the helical content of Nt17-pT3 peptides over different concentrations. The effect of oxM8 was stronger when it was added to the Nt17-AcK6 peptide; it significantly decreased its helical content over the whole range of tested concentrations (Supplementary Figure 5B). Taken together, these results suggest that the oxidation of M8 decreases the helicity of the Nt17 in the presence of PTMs, and this can play a role in modulating the aggregation of mutant Httex1.

To better understand how oxM8 and other PTMs influence the conformational properties of Nt17, we conducted atomistic molecular dynamics (MD) studies on Nt17. For these studies, we decided to include the first two glutamine residue as preliminary studies suggested that they could be involved in stabilizing Nt17 conformations. Previously, it has been shown that there is no difference in the overall structure between the Nt17 and the Nt19 (Nt17 + QQ) (Chiki et al., 2017). We simulated the Nt19 with single and double PTMs to analyze the crosstalk between oxidation at M8, and phosphorylation at T3 or S13, or acetylation at K6. The simulations were run for a total of 13 μs using the CHARMM36m force field and a modified TIP3P water model (Jorgensen et al., 1983), which have been shown to be effective for the simulation of intrinsically disordered proteins. The phosphate group of phosphorylated threonine was in a dianionic form. The parameters for phosphorylated threonine and acetylated lysine were obtained from the CHARMM36 parameters for modified residues (see Methods).

**FIGURE 4:**
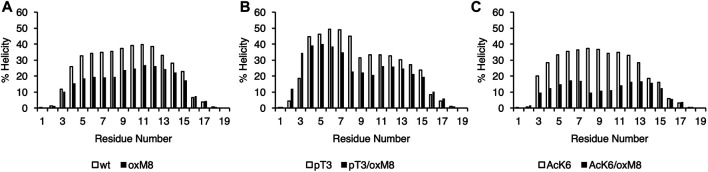
Helical propensity of Nt19. The secondary structure was obtained with STRIDE from the MD trajectories of **(A)** WT and oxM8 **(B)** pT3 and pT3/oxM8 **(C)** AcK6 and AcK6/oxM8. The helicity for each residue was calculated based on the sum of the total number of frames that are in *a*-helix or 3_10_ helical conformations divided by the total number of frames.

Both CD and MD showed that oxidation at M8 decreased the overall helicity of Nt19 Nt19-pT3 and Nt19-AcK6 (Table 1). The CD experimental data showed that for Nt19-pT3, oxidation at M8 resulted in a decrease of overall helicity by 13%, while a more significant effect was observed for Nt19-AcK6, whose helicity decreased by 69% (Table 1). Likewise, the MD results also showed that oxidation at M8 decreased the overall helicity of Nt19, with a more significant decrease in helicity for Nt19-AcK6 (Table 1). The residue-wise helicity calculated from MD showed that the core region of Nt19 (residue 4–13) had a more significant decrease, while the N-term residues (1–3) were less affected (Figure 4). Overall, oxM8 has a higher impact on Nt19-AcK6 and on the core region of Nt19 in general. These observations are consistent with our aggregation studies and CD analysis data.

**TABLE 1:** **Helicity of Nt19.** CD and MD both showed decrease in helicity when M8 was oxidized.

Nt19	WT *vs.* oxM8	pT3 *vs.* oxM8/pT3	AcK6 *vs.* oxM8/AcK6
**MD % change in helicity**	−20	−21	−60
**CD % change in helicity**	NA	−13	−69

### The Formation of a Short N-Terminal Helix in the Simulations Correlates With Suppressed Aggregation *In Vitro*.

**FIGURE 5:**
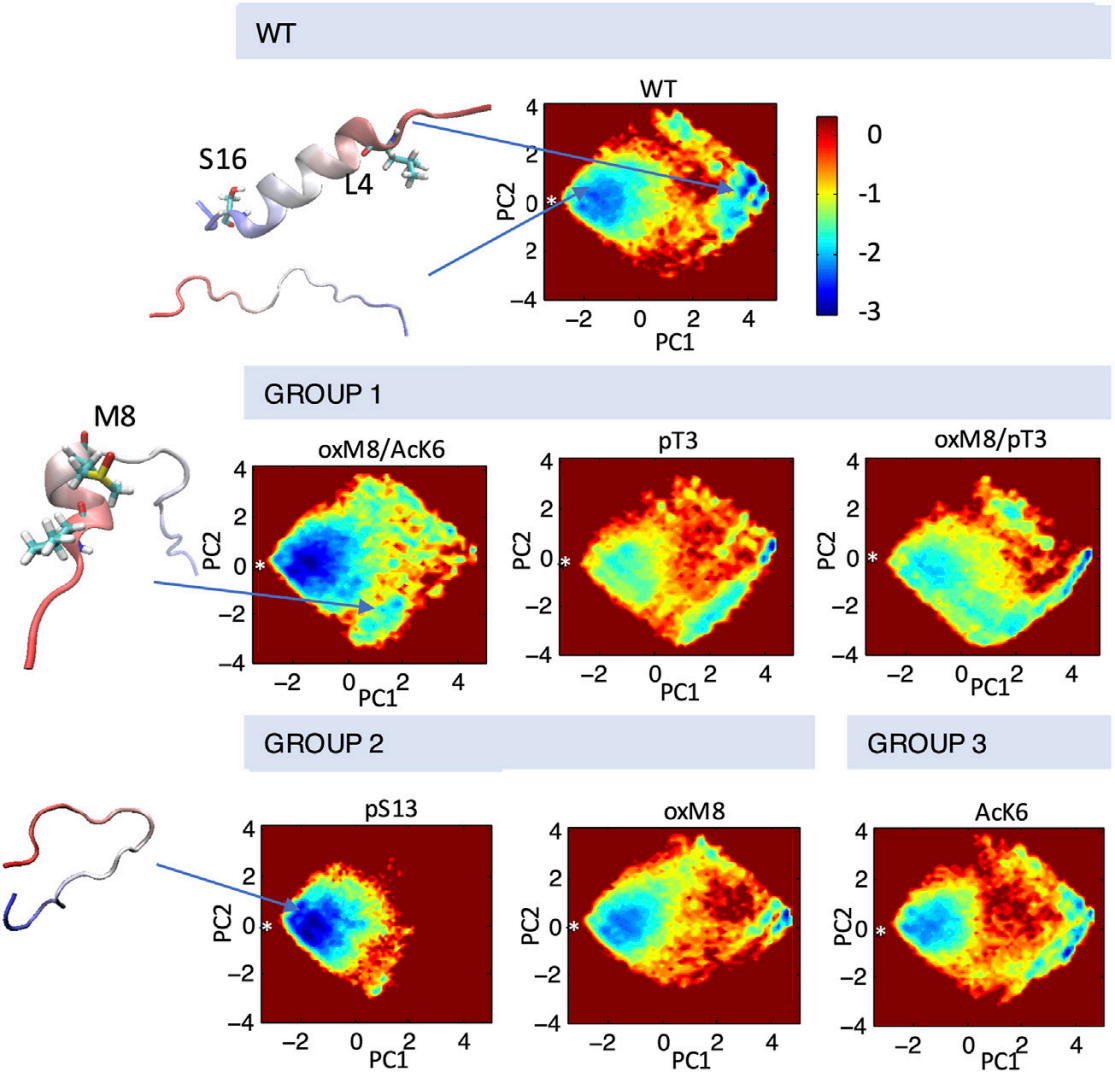
Conformational ensembles of Nt19 with different PTMs. dPCA was performed on dihedral angles calculated for the Nt19 backbone, and a free energy map was generated with the first two components. The white asterisk indicated the starting structure (in extended conformation). The major structures that correspond to local minima include fully disordered conformation, short N-term helix, and long helix. The peptides are classified based on the effect of each PTM on helicity and aggregation rate: oxM8/Ack6, pT3, and oxM8/pT3 increased Nt19 helicity and decreased aggregation rate (GROUP 1), pS13 and oxM8 decreased Nt19 helicity and decreased aggregation rate (GROUP 2), AcK6 had no significant effect on aggregation rate (GROUP 3).

The MD results were further analyzed to explore the structure and dynamics of PTM crosstalk and gain insight into the structural basis underlying the differential effects of Nt17 PTMs on the aggregation of mutant Httex1. We first performed a principal components analysis of the backbone dihedral angles (dPCA) (Mu et al., 2005) to distinguish among the various conformations adopted by the peptides with different PTMs. The first two components that depict a large part of the differences in the conformations were used for generating an energy map (Figure 5). dPCA showed well-separated minima corresponding to various helical forms and revealed a very broad and shallow minimum corresponding to a wide-range of unfolded conformations connected by very low barriers.

**FIGURE 6:**
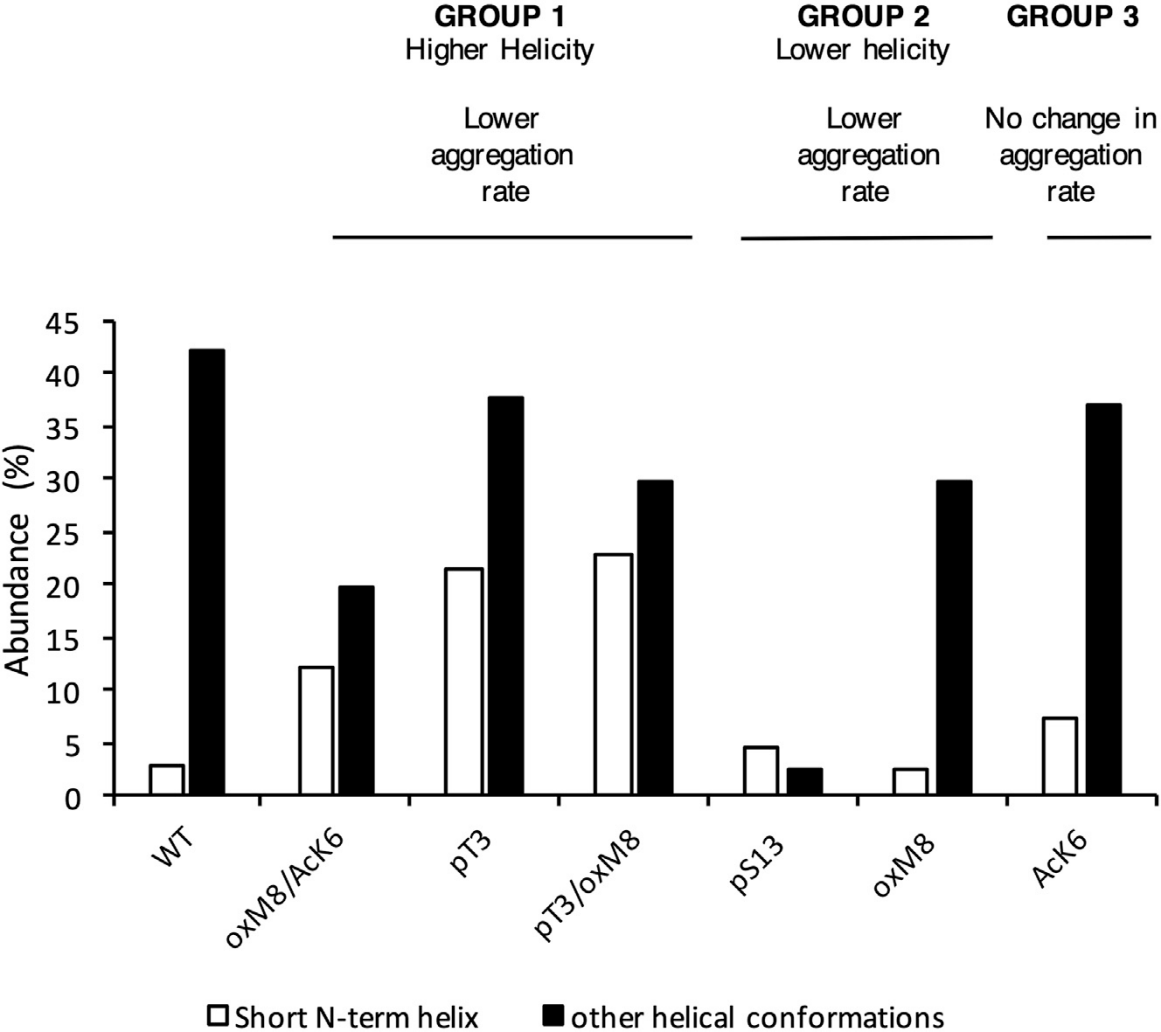
The abundance of N-term helix was high for PTMs that delayed aggregation. The abundance of short N-terminal helix and other helical forms was calculated for each peptide. Peptides with a lower aggregation rate showed a higher abundance of N-terminal helix and a lower abundance of other helical conformations.

To investigate the connection between the major conformations and the aggregation rates of mHttex1 with different PTMs, we classified the PTMs into three groups based on their effects on the aggregation of mHttex1 and the helicity of Nt17. In Group 1, we grouped PTMs that showed higher helicity and had a lower aggregation rate. In Group 2, PTMs that showed lower helicity but also lower aggregation rate, while in Group 3, PTMs that showed an aggregation rate similar to unmodified Httex1. dPCA showed that for all PTMs in Group 1, there were several local minima corresponding to conformations where the first 8 N-terminal residues adopted a helical conformation while the rest remained disordered (Figure 5). When T3 was phosphorylated, and when both T3 and oxM8 modifications were present, this short N-terminal helix was 7 times more populated than for the unmodified peptide. Meanwhile, the N-terminal helix was 4 times more populated in oxM8/AcK6 compared to the unmodified peptide, yet the abundance of all other helical forms was reduced by half compared to the unmodified peptide (Figure 6). For Group 2, the abundance of the N-terminal helix of pS13 and oxM8 was similar to the unmodified peptide, but a 94 and 30% decrease was observed for other types of helices in pS13 and oxM8. For Group 3, the abundance of either short N terminal helix or other types of helices in AcK6 was similar to that of the unmodified peptide. By comparing the helicity data with aggregation data, it then appears that the higher abundance of a short N-terminal helix and lower abundance of other Nt17 helical conformations, like a long helix or short C-terminal helix in the MD simulations, correlates with lower aggregation rates of mHttex1.

### Oxidation of M8 Increased Abundance of the Short N-Terminal Helix

To analyze how the PTMs modulate the stability of the short N-terminal helix, we searched for the key differences in structural dynamics in simulations of peptides with oxidized and reduced methionine. For this, we computed the distance matrix between Cα atoms for all frames of each MD simulation (Supplementary Figure 6), compared the distributions of Cα pairwise distances through Kullback-Leibler divergences of two distance matrices, as described in Methods, and plotted the divergences into heat maps. Heat maps comparing pT3 with WT and oxM8/pT3 with WT (Supplementary Figure 6) show a significant change in the distance between the first eight residues and residue 1 or 2. Heat maps comparing WT and oxM8/AcK6 show that significant changes occur at a distance between residue 4 and residue 9/10.

**FIGURE 7:**
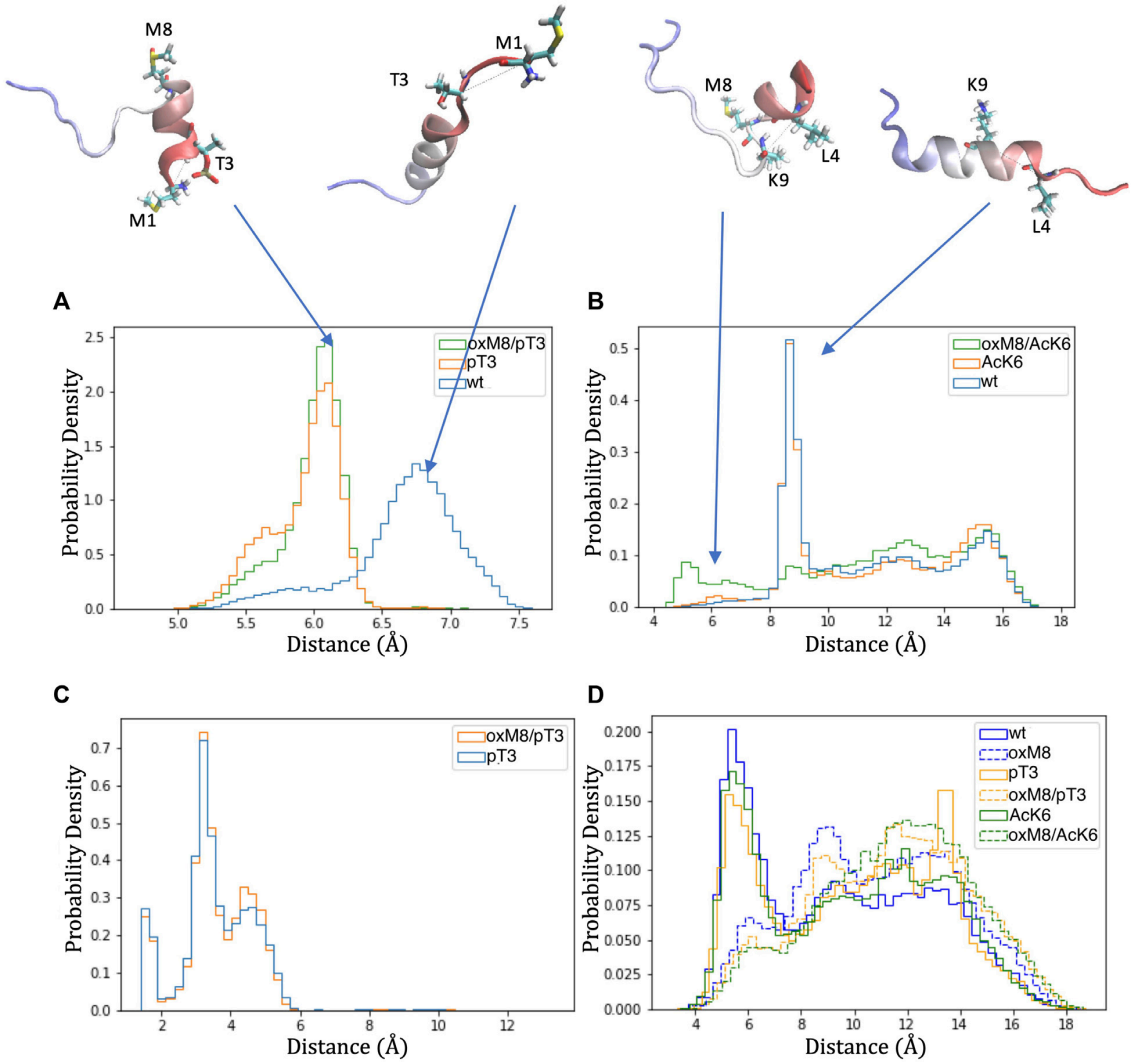
Distance distribution of atom pairs in Nt19 with different PTMs. **(A)** Distance between Cα of M1 and Cα of T3 was lower in pT3 and oxM8/pT3 than in WT. **(B)** The distance between Cα of L4 and K9 was lowered in oxM8/AcK6 compared to WT and AcK6 due to the bend at M8. **(C)** A peak below 4 Å was shown in the distribution of the distance between hydrogens of NH_3_^+^ and oxygens of the pT3 phosphate group, indicating pT3 might promote ionic interaction at this N-terminal end. **(D)** The distance between the sulfur atom of M8 and the center of the F11 aromatic ring increased in peptides with M8 oxidation, indicating the stabilization by the interaction of sulfur and the aromatic ring was weakened.

The distance distributions (Figure 7A) are similar for pT3 and pT3/oxM8 but were different for WT. The peak for oxM8/pT3 and pT3 corresponds to a conformation where the phosphate group of pT3 forms a salt bridge with the NH_3_^+^ group of M1 (Figure 7C), which is not observed in WT, where M1 and T3 remained apart. A previous study by Yalinca et al. has shown that phosphorylation stabilizes helical conformation between A2 and L7, because of the increased negative charge that counterbalances the helix dipole (Yalinca et al., 2019). Also, the distribution of distances between Cα of L4 and K9 in WT, AcK6, and oxM8/AcK6 (Figure 7B) is similar for WT and AcK6 but different for AcK6/oxM8. The peak for WT and AcK6 corresponded to the distance between L4 and K9 when they were in a helix conformation. The peak for AcK6/oxM8 corresponds to a conformation where there was a turn at M8, so the distance between L4 and K9 decreased.

Another aspect we examined is the distance between the sulfur atom of M8 and the center of the aromatic ring of F11 because a previous study (Valley et al., 2012) has shown that when this distance is below 6 Å, an interaction between sulfur of M8 and the aromatic ring could stabilize the structure. For WT, pT3, and AcK6, a peak below 6 Å (Figure 7D) was observed for the distance between M8’s sulfur atom and F11’s aromatic ring. When M8 is oxidized, the distance distribution shifts toward larger values, suggesting the disruption of the stabilization effects brought by these interactions.

The residue pair distance analysis for WT, pT3, AcK6, oxM8/pT3, and oxM8/AcK6 shows that the change in abundance of short N-terminal helix and other helix resulted from 1) stabilization of N-terminal helix by phosphate group of T3 (pT3, oxM8/pT3) and 2) breaking the long helix between residue 8 and 9 with oxidation at M8 (oxM8/AcK6) and destabilization of the long helix due to disruption of the interaction between residue 8 and 11.

## Discussion

Among all Htt Nt17 PTMs, methionine oxidation remains the least well understood and studied. To address this knowledge gap, we first developed an efficient method to produce milligram quantities of highly purified untagged mutant Httex1 that is site-specifically oxidized at M8. To investigate the crosstalk between M8 oxidation and neighboring PTMs, we used protein semisynthetic and chemoenzymatic strategies developed by our group (Chiki et al., 2017) to generate mutant Httex1 proteins bearing oxidized M8 and acetylated K6 (mHttex1-AcK6/oxM8) or phosphorylated at T3 (mHttex1-AcK6/pT3). These advances enabled us to investigate, for the first time, the role of crosstalk between these different PTMs in regulating Nt17 conformation and mutant Httex1 aggregation *in vitro*. We choose K6 and T3 because of their close proximity to M8 and the fact these are the only two PTMs that have been shown to significantly influence the helicity of Nt17 and modify the effect of neighboring PTMs on Nt17 structure and Httex1 aggregation (Chiki et al., 2017).

Our results demonstrate that oxidation at M8 delayed the aggregation of mHttex1 but did not alter the morphology of the structure of the Httex1 fibrils. We hypothesized that this delay is caused by methionine oxidation-induced formation of oligomers at the early stages of the aggregation, which undergo relatively slower conversion to fibrils. As shown in Figure 3 and highlighted in previous studies (Vieweg et al., 2016; Chiki et al., 2017; DeGuire et al., 2018), oligomers are rarely observed during the fibrillization of the unmodified mutant Httex1 due to its high propensity to misfold and fibrillize. In contrast, our imaging studies of oxM8 aggregation consistently revealed the transient accumulation of oligomers at the early stages of the aggregation process (Supplementary Figure 3).

Although acetylation at K6 was previously shown not to influence the aggregation of mutant Httex1, when this modification is combined with oxidation at M8, we observed a strong inhibitory effect on mutant Httex1 aggregation and the accumulation of mainly oligomeric and short fibrillar structures. In contrast, the combination of methionine oxidation and phosphorylation at T3 did not modify the aggregation inhibitory effect induced by pT3. Our findings are consistent with previous observations on the effect of methionine oxidation on the aggregation of other amyloid-forming proteins. For example, oxidation at M35 attenuates the aggregation amyloid-β(1–40) (Friedemann et al., 2015; Gu and Viles, 2016), Aβ1-42, and the highly amyloidogenic Arctic Aβ1-40 variant (Johansson et al., 2007). Similarly, oxidation of methionine 109 and 112 in the prion peptide PrP106-126 inhibits its fibrillization *in vitro* (Bergström et al., 2007). Furthermore, methionine oxidation was shown to inhibits the aggregation of alpha-synuclein protein and promote the formation of stable oligomers (Zhou et al., 2010). In the case of Httex1, previous studies have shown that oxidation at M8 abolished aggregation of a model peptide consisting of Nt17 plus ten glutamine residues (Nt17Q10) (Ceccon et al., 2018).

To understand the structural basis underlying the effect of methionine oxidation and its crosstalk with pT3 and AcK6 on mHttex1, we performed CD and MD analyses of the conformational properties of the unmodified Nt17 peptide and Nt17 peptides bearing pT3, or AcK6 in the presence or absence of the oxM8. We observed that methionine oxidation reduced the helical content of Nt17, independent of co-occurring PTMs and peptide concentration. Consistent with our previous studies (Chiki et al., 2017; DeGuire et al., 2018), we observed that the effect of PTMs on the overall helicity of the Nt17 peptide did not correlate with their impact on the aggregation propensity of mutant Httex1 *in vitro*. However, it was noticed that all PTMs that are associated with a slower aggregation rates have a higher abundance of short helices at the first 8 N-term residues and a lower abundance of other helical conformations. According to the simulations, the higher abundance of short N-term helices in Nt17-pT3 and oxM8/pT3 could derive from direct stabilization via a salt bridge between the phosphate group at T3 and the NH_3_^+^ group of M1, while their higher abundance in Nt17-oxM8/AcK6 could result from breaking long helices with a turn between residue 9 and 10, as well as destabilization of the long helices due to disruption of the interaction between residue 8 and 11 of oxM8/AcK6. The simulation results provide an interesting perspective, i.e., that the overall helicity only provides ensemble-averaged structure information; with the same overall helicity as measured by CD, some modified peptides contain a higher abundance of short N-term helix, and some contain a higher abundance of the long helix or short C-term helix forms. Previous studies suggested that the helical conformation of Nt17 is a major driver of the initiation of mutant Htt oligomerization and fibrillization. Our findings suggest Nt17 exists as an ensemble of different helical conformations, among which some might be vital for aggregation, while others, like short N-term helix, have no impact or even disfavor the aggregation process.

**FIGURE 8:**
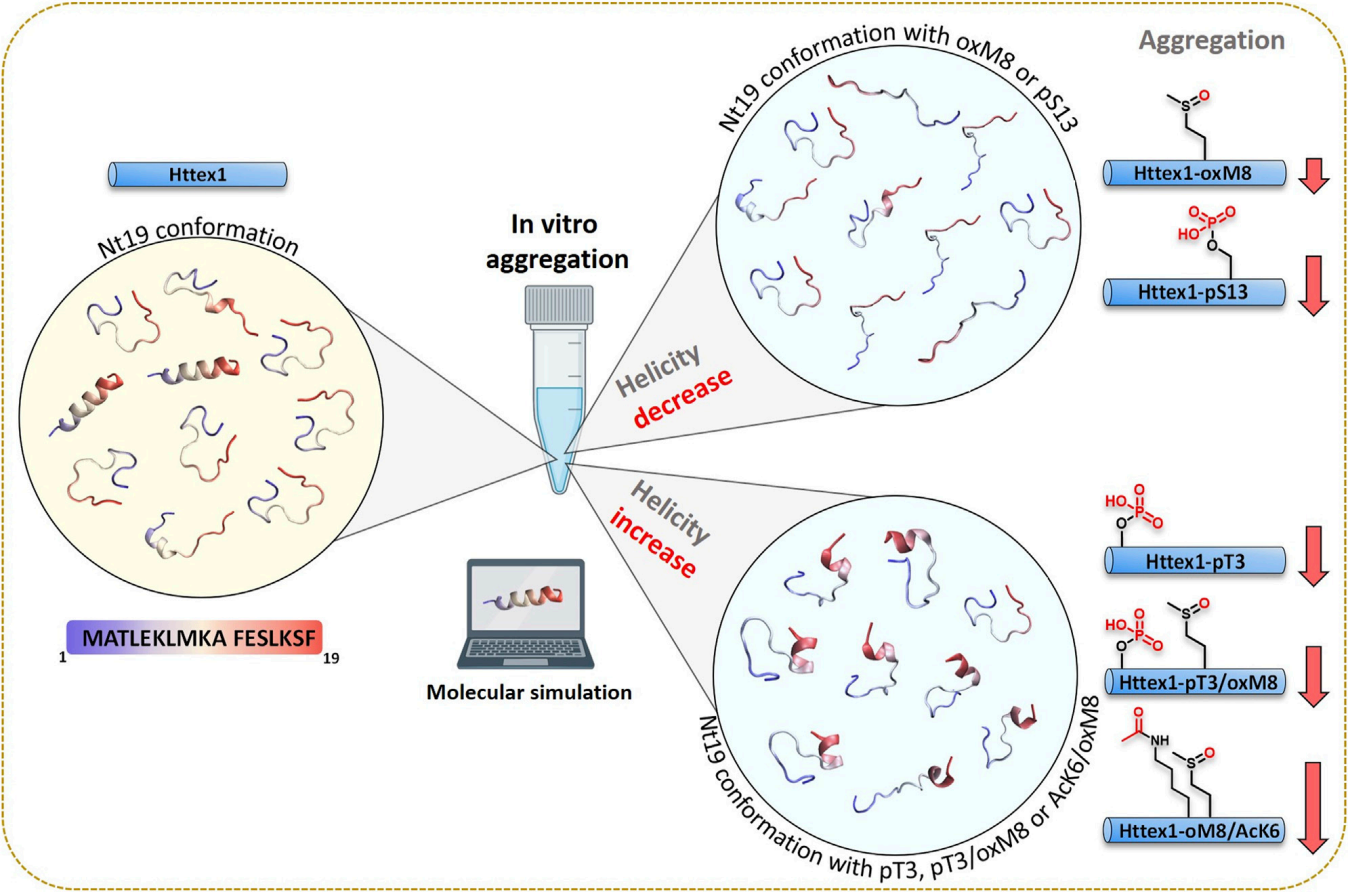
A combined experimental and computational approach unravels the structural basis of PTM crosstalk in regulating aggregation of Huntingtin exon 1.

Our findings on the effect of the crosstalk between the different Nt17 PTMs highlight the complexity of the Nt17 PTM code and suggest that Htt’s normal function and aggregation are likely regulated by a complex interplay between different PTMs (Figure 8). A major challenge in addressing this complexity is the large number of possible PTM combinations, even within short stretches of sequences in proteins, such as Nt17. This, combined with the challenges of introducing multiple PTMs into proteins, has led to either abandoning efforts to investigate this complexity or resorting to studying protein fragments. Our findings here show that while working with peptide fragments provides useful insights, extrapolations of findings from these fragments to predict their properties in the context of full-length proteins are not straightforward. The work presented here represents our initial efforts in this direction and aims at exploring the extent to which this is possible using peptide systems in which the effect of PTMs can be experimentally and computationally measured and predicted. Also, our work demonstrates the promising potential of integrating experimental and computational approaches for characterizing molecular mechanisms.

## Materials and Methods

### Materials

The pTWIN1 vector containing human Httex1 fused to His6-SUMO was ordered from GeneArt Gene Synthesis (Life Technologies); *E. coli* B ER2566 from NEB; ampicillin, DTT, isopropyl *ß*-D-1-thiogalactopyranoside (IPTG), and hydrogen peroxide solution 30%, imidazole, complete Protease Inhibitor Cocktail, magnesium chloride (MgCl_2_), magnesium sulfate (MgSO_4_), and trifluoroacetic acid from Sigma; PMSF from AppliChem; EGTA solution from Boston Bioproducts; Mg-ATP from Cayman; EDTA from Fisher Scientific; Luria Broth (Miller’s LB Broth) from Chemie Brunschwig; acetonitrile (HPLC-grade) from Macherey Nagel; spectrophotometer semi-micro cuvettes from Reactolab; C4 HPLC column from Phenomenex; HisPrep 16/10 column from GE Healthcare; GCK kinase (0.5 μg/ul, cat. # BC047865) from MRC PPU Reagents; Uranyl formate (UO2(CHO2)2) and Formvar/carbon 200 mesh, Cu 50 grids from EMS; high-precision cell made of Quartz SUPRASIL 1 mm light path from Hellma Analytics; Dulbecco’s Buffer Substance (PBS w/o Ca and Mg) ancienne ref. 47,302 (RT) SERVA from Witech; and 100-kD Microcon fast flow filters from Merck Millipore.

### Expression and Purification of SUMO-Httex1-43Q

Expression of His6-SUMO-Httex1-Qn (*n*= 23 or 43). The expression and purification of His-SUMO-Httex1-43Q were performed as previously reported (Reif et al., 2018). Httex1-43Q with a His-SUMO tag at its N-terminal was cloned into the pTWIN1 plasmid with ampicillin (Amp) resistance. Then, the plasmid was transformed in *E-coli* ER2566, and the resulting transformed bacterial cells were plated onto an agar plate containing ampicillin (Froger and Hall, 2007). Next, 400 ml LB + Amp (100 μg/ml) medium was inoculated with a single colony and incubated at 37°C overnight to start the day after the 12L expression at an optical density (OD_600_) density of 0.15. When the OD_600_ value was around 0.6, the culture was induced with 0.4 mM IPTG and incubated at 18°C overnight. The cells were harvested by centrifugation (3,993 rpm, 4°C, 10 min) and resuspended in buffer A (50 mM Tris, 500 mM NaCl 30 mM Imidazole, pH 7,5, 0,65 um filtrated) with PMSF and protease inhibitors. The suspended bacterial pellet was subjected to sonication on ice (70% amplitude, total sonication time 5 min, intervals of 30 s sonication, 30 s pause), followed by centrifugation (39,191 rpm, 4°C, 60 min). The supernatant was filtered (0.45 µm, syringe filters) and passed through a Ni-NTA column. The protein was then eluted with 100% IMAC buffer B (50 mM Tris, 500 mM NaCl 15 mM Imidazole, pH 7.5, 0.65 µm filtrated). The purified fusion protein was kept ice for further reactions. The final yield of Httex1-43Q was 1.23 mg/L of culture.

### Production of Httex1-43Q oxM8 and Httex1-43Q oxM8/pT3

SUMO-Httex1-43Q (10 mg in 18 ml) fusion protein was treated with 1 ml of 30% H_2_O_2_ to perform the oxidation directly on the SUMO fusion protein. After 2 h of the reaction, 30 µl was supplemented with 1 µl of ULP1 (1 mg/ml), and the extent of the oxidation was verified by LC/MS. 1 oxidation corresponds to +16 Da to the molecular weight of Httex1-43Q. When the completion of the oxidation was confirmed, 400 µL of ULP1 enzyme was added to the reaction solution to cleave the SUMO tag, and the reaction was directly injected in RP-HPLC, C4 column using a gradient of 25–35% solvent B (Acetonitrile +0.1 TFA) in solvent A (H_2_O+ 0.1% TFA), over 40 min at 15 ml/min. The fractions were analyzed by LC/MS; the ones containing the protein were pooled and lyophilized. The quality of the of protein was analyzed using LC/MS, UPLC, and SDS-PAGE. The final yield of Httex1-43Q oxM8 was 0.76 mg/L of culture.

For the generation of Httex1-43Q oxM8 and Httex1-43Q oxM8/pT3, the oxidation was performed as above, and when completed, the oxidized fusion protein was dialyzed against 4 L of TBS buffer overnight at 4°C and then supplemented with 10x phosphorylation buffer to obtain a final buffer with the following composition and concentration: 50 mM Tris, 25 mM MgCl_2_, 8 mM EGTA, 4 mM EDTA, 1 mM DTT. Mg-ATP (5 mM) was added to the solution, and the pH was adjusted, and finally, GCK was added at a ratio of 1:30 w/w (kinase:protein) to the Httex1-43 oxM8 (666 µL), and the reaction was incubated at 30°C overnight. Next, the extent of the phosphorylation was verified by LC/MS, and when it was completed, 400 µL of ULP1 was added to the phosphorylation reaction solution to cleave the SUMO tag. Finally, Httex1-43Q-oxM8 was separated from the SUMO tag, and other impurities by HPLC and the fractions containing the protein were pooled and analyzed by LC/MS, UPLC, and SDS-PAGE. The final yield of Httex1-43Q oxM8/pT3 was 0.6 mg/L of culture.

### Semi-Synthesis of Httex1-43Q oxM8/AcK6

The semi-synthesis of Httex1-43Q oxM8/AcK6 was performed as previously reported (Chiki et al., 2017). Briefly, 5 mg of Htt-A10C-90-43Q with free N-terminal cysteine was dissolved in neat TFA, and the TFA was dried under nitrogen after 30 min. The dried Htt-A10C-90-43Q was redissolved in 5.0 ml ligation buffer (8 M urea, 0.5 M l-Proline, 30 mM D-Trehalose, 100 mM TCEP and 100 mM MPAA). The pH was adjusted to 7.0 with 10 M NaOH, and the peptide Ac-2-9Nbz AcK6/oxM8 peptide (2.5 mg) was added to the reaction mixture. The native chemical ligation was monitored by LC-ESI-MS until the complete consumption of A10C-90 43Q. Next, the ligation reaction was desalted with hitrap 26/10 desalting column, and fractions containing the Httex1-A10C-43Q oxM8/pT3 were pooled and lyophilized. To recover the native alanine, the protein mixture was disaggregated and dissolved in 100 mM TCEP, 40 mM L-methionine, 20 vol% acetic acid in H_2_O. Freshly prepared nickel boride suspension (1.0 ml) was added to the resulting solution, and the resulting suspension was incubated at 37°C. After 2 h, a loss of 32 Da was observed. Insoluble nickel was removed from the reaction mixture via sedimentation (4°C, 4,000 g, 10 min). The supernatant was subjected to RP-HPLC purification with a gradient of 25–55% buffer B (MeCN +0.1% TFA) in buffer A (H_2_O+ 0.1% TFA). The fractions containing Ac-Httex1-43Q oxM8/pT3 were analyzed by LC-MS and UPLC, pooled, and lyophilized. The final purity of the protein was checked by LC/MS, UPLC, and SDS-PAGE. The final of the semisynthesis reaction was 27% (1.35 mg of final protein/5 mg of Htt-A10C-90-43Q starting material).

### UPLC-Based Sedimentation Assay

To perform aggregation studies, Httex1 was disaggregated as previously reported (Reif et al., 2018) using TFA, and after evaporating the TFA, the protein was filtered through a 100-kDa filter. The final volume was adjusted with PBS to have the desired concentration and incubated at 37°C. To determine the percentage of the remaining soluble monomer during the aggregation, 40 µL was taken at each indicated time point, and insoluble aggregates were removed by centrifugation (4°C, 20,000 g, 30 min). The supernatant was injected into the UPLC. The peak area was measured for each time point, and the changes in the area were used to calculate the fraction of soluble protein compared to t = 0 being 100%. The exponential decay function $y=Ae^{\;b\left(x\right)}+c$ was used to fit the data of different aggregation curves (O'Nuallain et al., 2006b). All the mutant Httex1 aggregated protein curves had an adjusted *R* ^2^ ranging from 0.97 to 0.99 (except Httex1-43Q AcK6/oxM8 with *R* ^2^ = 0.78).

### Electron Microscopy

For TEM analysis, 5 µl of aggregation solution was spotted onto a Formvar/carbon-coated 200-mesh glow-discharged copper grid for 1 min. The grid was then washed 3x with water and stained for 3×10 s with 0.7% w/v uranyl formate. Imaging was performed on a Tecnai Spirit BioTWIN electron microscope equipped with a LaB6 gun and a 4 K × 4 K FEI Eagle CCD camera (FEI) and operated at 80 kV.

### Atomic Force Microscopy (AFM) Imaging

AFM was performed on freshly cleaved mica discs that are positively functionalized with 1% (3-aminopropyl)triethoxysilane (APTS) in an aqueous solution for 3 min at room temperature. For fibril deposition on the substrates, a 20-µL aliquot of 5.2 µM protein solution was loaded on the surface of mica discs at defined time-points. Deposition took 3 min and was followed by a gentle drying by nitrogen flow. The mica discs were stored in a desiccator for one day before imaging to avoid prolonged exposure to atmospheric moisture. AFM imaging was performed at room temperature by a Park NX10 operating in a true non-contact mode that was equipped with a super sharp tip (SSS-NCHR) Park System cantilever. The length and height quantifications were performed semi-automatically using XEI software developed by Park Systems Corp by taking advantage of the grain detection method and thresholding (masking) the background values. The quantified data was plotted and analyzed in GraphPad Prism 8.

### Circular Dichroism

mHttex1 proteins (100 µL) during the aggregation or 120 µL of the Nt17 peptides (at 60 µM in PBS) were removed from the mixture and analyzed using a Jasco J-815 CD spectrometer and a 1.0 mm quartz cuvette. Ellipticity was measured from 195 to 250 nm at 25°C.

Origin was used to smooth the data through Savitzky-Golay fitting (8 points window). The mean residue molar ellipticity (θMRE) was plotted, and BeStSel (Micsonai et al., 2018) was used for fitting and calculating the helicity. Average helicity and standard deviation were calculated from 3 repeats.

### Molecular Dynamics Simulations

The starting structure for molecular dynamics simulations was a fully extended form of Htt 19 peptide. The length of this extended conformation was 68 Å, and the buffer distance between each side of the peptide and the periodic boundary was 10 Å, resulting in a water box with a width of 88 Å. The HTT 19 peptide was solvated using explicit CHARMM36m (Huang et al., 2017) modified TIP3P water model (Jorgensen et al., 1983) and 20 mM K^+^ and Cl^−^ ions. The simulation was conducted with GROMACS (Abraham et al., 2015), and CHARMM-GUI was used to generate inputs. A CHARMM36m forcefield was used with modified residues for phosphorylated threonine and acetylated lysine residues (Grauffel et al., 2010). The phosphate group of phosphorylated threonine was in a dianionic form. The oxidized methionine used in this simulation was an R diastereomer, and its parameters were obtained from previously published papers (Lockhart et al., 2020). The system was minimized with the steepest descent and equilibrated to 1 atm and 303.15 K with constraints on the backbone. The nonbonded interaction cut-off was 12 Å. The time step was 1 fs for equilibration and 2 fs for production. The Nosé−Hoover temperature (Nosé, 1984) coupling method was used to maintain temperature, and the isotropic Parrinello−Rahman (Parrinello and Rahman, 1981; Nosé and Klein, 1983) method was used for pressure coupling. LINCS algorithm (Hess et al., 1997) was used for H-bond. The production was run for 13 μs for each system.

### Data Analyses

To perform dPCA (Mu et al., 2005), the dihedral angles were calculated for the Nt19 backbone, and a transformation from the space of dihedral angles (ϕ_n_, ψ_n_) to metric coordinate space was done by taking trigonometric function (cos ϕ_n_, sin ϕ_n,_ cos ψ_n,_ sin ψ_n_). Then, the transformed dihedral data of each post-translationally modified Nt19 was combined, and a PCA analysis was done. A free energy map was generated with the first two components. The secondary structure was obtained with the STRIDE (Frishman and Argos, 1995) algorithm implemented in VMD (Humphrey et al., 1996). The overall helicity for each peptide was calculated based on the sum of the total number of residues that are in *a*-helix or 3_10_ helical conformations at every frame and then divide by the total number of frames.

We analyzed and compared the effects of each post-translational modification of Nt1-19 on the dynamics using a statistical analysis of the distribution of pairwise distances between residues. For each MD of Nt1-19, we reduce the ensemble of conformations to a statistical description of the distances between all pairs of Cα in Nt1-19. For each pair of residues within Nt1–19, we compute the distribution of the Euclidean distances between the two Cα with a bin resolution of 0.5 Å. We, therefore, describe every MD with a corresponding histogram of distances summarizing the dynamics of the structure. The differences in residue-residue dynamics between MDs can be quantified using the Kullback-Leibler divergence (Kullback and Leibler, 1951) from a reference histogram of distances.

## Data Availability

The raw data supporting the conclusions of this article will be made available by the authors, without undue reservation.
