# Supplementary material for: Investigating Crosstalk Among PTMs Provides Novel Insight Into the Structural Basis Underlying the Differential Effects of Nt17 PTMs on Mutant Httex1 Aggregation
Source: Front Mol Biosci. 2021 Jul 26;8:686086. doi: 10.3389/fmolb.2021.686086 (PMC8352439; doi:10.3389/fmolb.2021.686086)
Supplement: Supplementary file 1 [file DataSheet1.pdf]

Supplementary Material

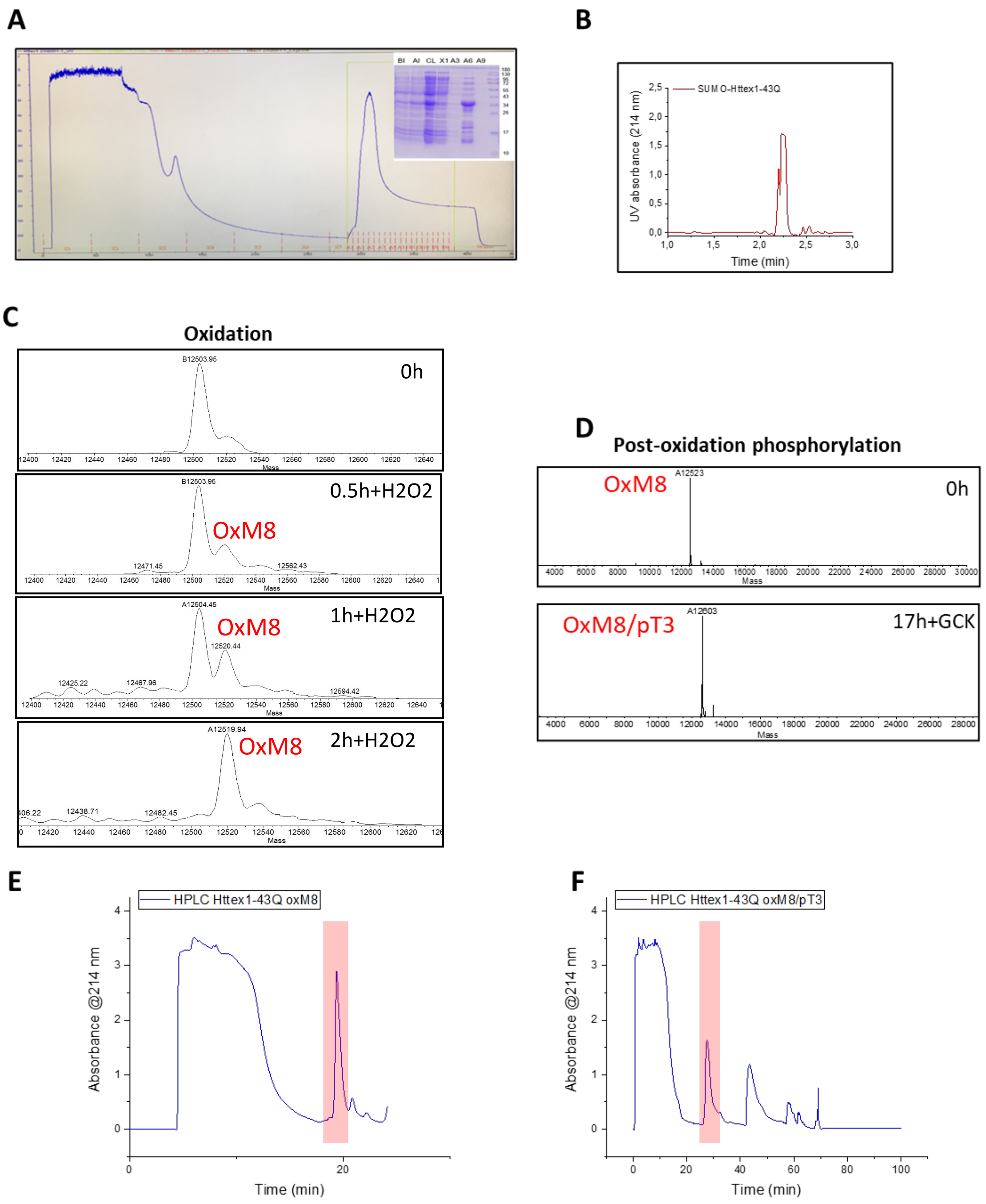

Figure S1. (A) Representative chromatogram of the IMAC purification of SUMO-mHttex1 and the analysis by SDS-PAGE of the purification fractions. **(B)** Analysis by UPLC of the fusion SUMO-mHttex1 after IMAC purifications. **(C)** Monitoring by ESI/MS of SUMO-mHttex1 oxidation by H_2O_2 overtime after an analytical SUMO tag cleavage by ULP1. **(D)** Monitoring of SUMO-mHttex1 phosphorylation by GCK after an analytical SUMO tag cleavage by ULP1. **(E-F)** RP-HPLC chromatograms for the purification of mHttex1 oxM8 (E) and mHttex1 oxM8/pT3 (F). (protein of interest is highlighted in red).

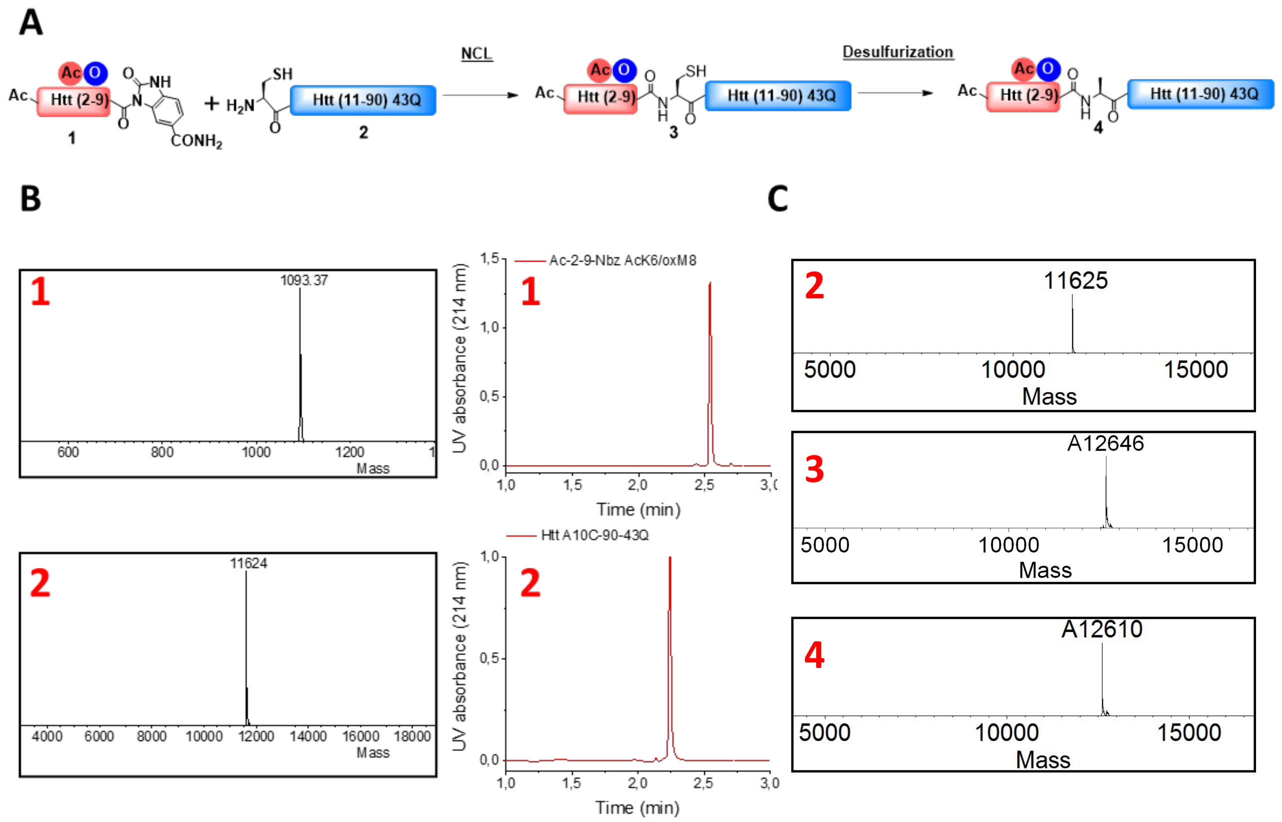

Figure S2. (A) Schematic representation for the semisynthetic strategy used for the generation of mHttex1-oxM8/AcK6 **(4)** (adapted from [30]). **(B)** Characterization by ESI/MS and UPLC of Htt Ac-2-9-Nbz oxM8/AcK6 **(1)** and Htt A10C-90 43Q **(2)**, both are the starting material for the semi-synthesis. The native chemical ligation of **(1)** and **(2)** was performed in (8 M urea, 0.5 M L-Proline, 30 mM D-Trehalose, 100 mM TCEP, pH 7), and the ligation was monitored by ESI/MS **(C)**. When the NCL was completed, the reaction solution containing Httex1-43Q-oxM8/AcK6 A10C **(3)** was dialyzed and lyophilized and then desulfurized in 100 mM TCEP, 40 mM L-methionine, 20 vol% acetic acid in H_2O pH 1, the desulfurization of Cys to Ala was monitored by ESI/MS.

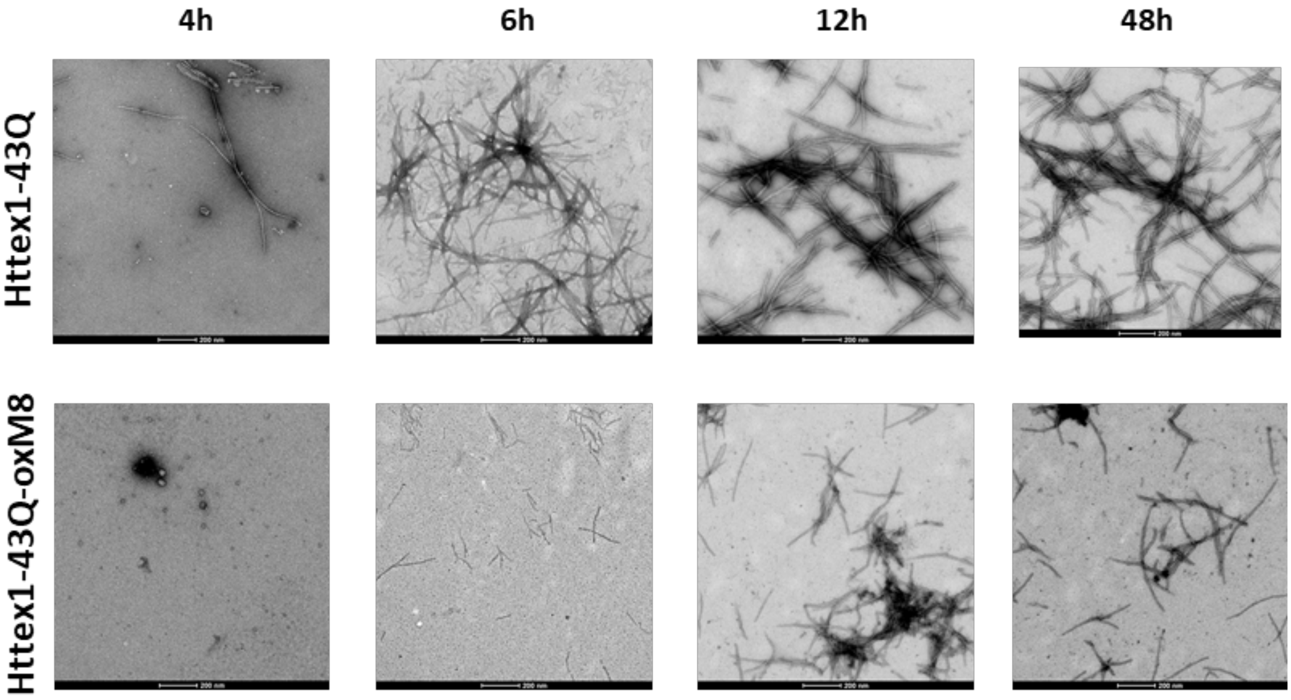

Figure S3. Over-time aggregation monitoring by electron microscopy of Httex1-43Q-oxM8 compared to unmodified Httex1-43Q (at 10 uM).

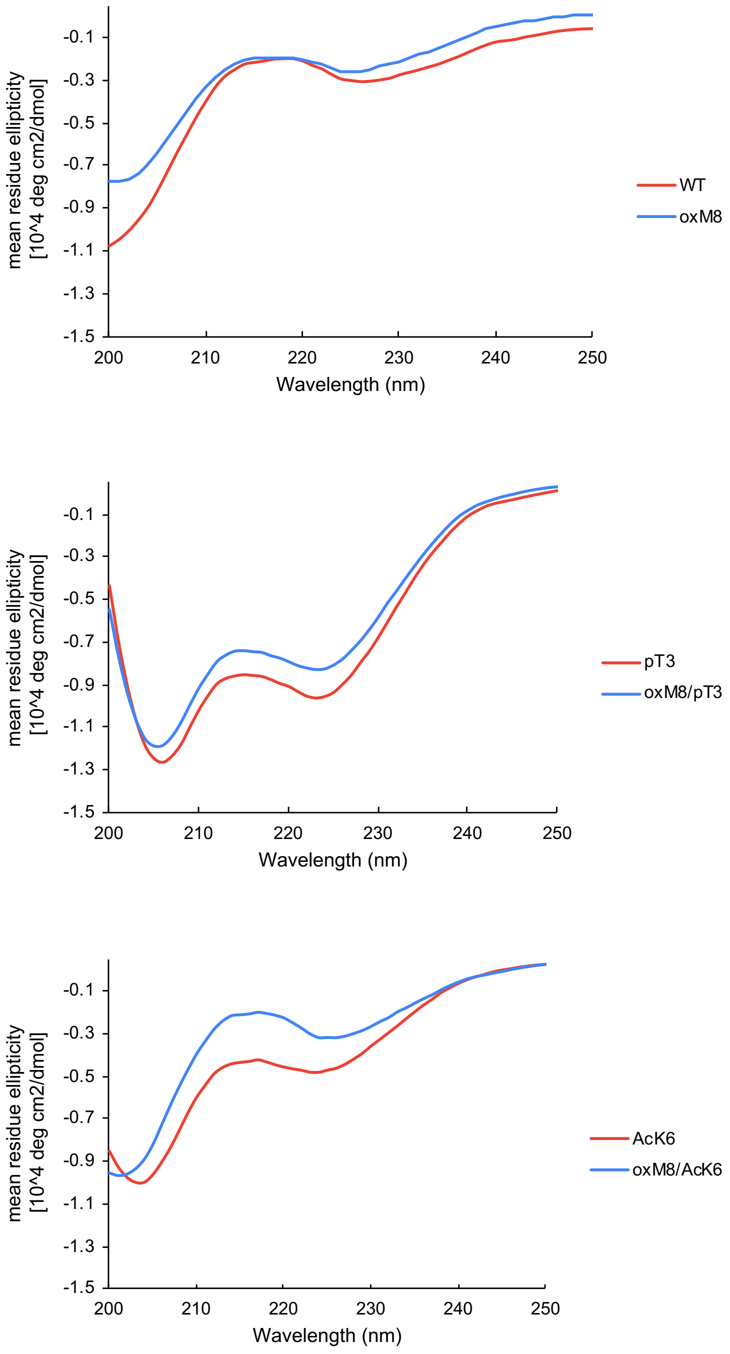

Figure S4. Far-UV CD spectra of Nt17-WT, and Nt17-oxM8, Nt17-AcK6, Nt17-AcK6/oxM8, Nt17-pT3 and Nt17-pT3/oxM8 at 60 µM.

Table S1: Helical content (%) calculated from 3 repeats for the different peptides at 60 μM.

**Nt17 peptide**	**CD Helical content (%)**
WT	0.1 ± 0.1
oxM8	1.7 ± 0.5
pT3	21.8 ± 0.7
pT3/oxM8	18.9 ± 0.5
AcK6	5.0 ± 0.2
AcK6/oxM8	1.6 ± 0.2

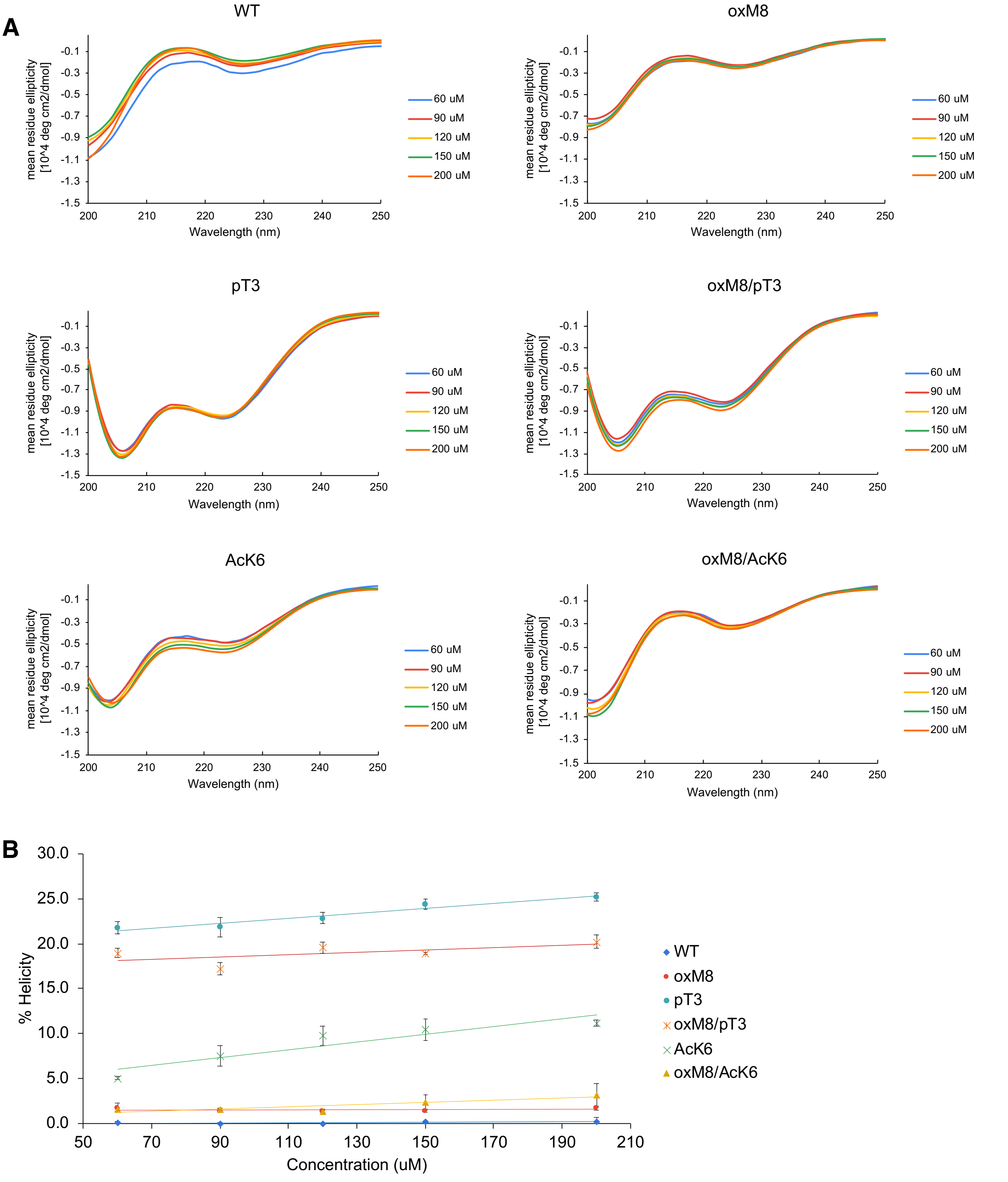

Figure S5. **(A)** Far-UV CD spectra of Nt17-WT, and Nt17-oxM8, Nt17-AcK6, Nt17-AcK6/oxM8, Nt17-pT3 and Nt17-pT3/oxM8 at 60, 90, 120, 150, and 200 µM. **(B)** Helical content for the peptides in the function of the different concentrations. The error bars indicate the standard deviations calculated from 3 repeats.

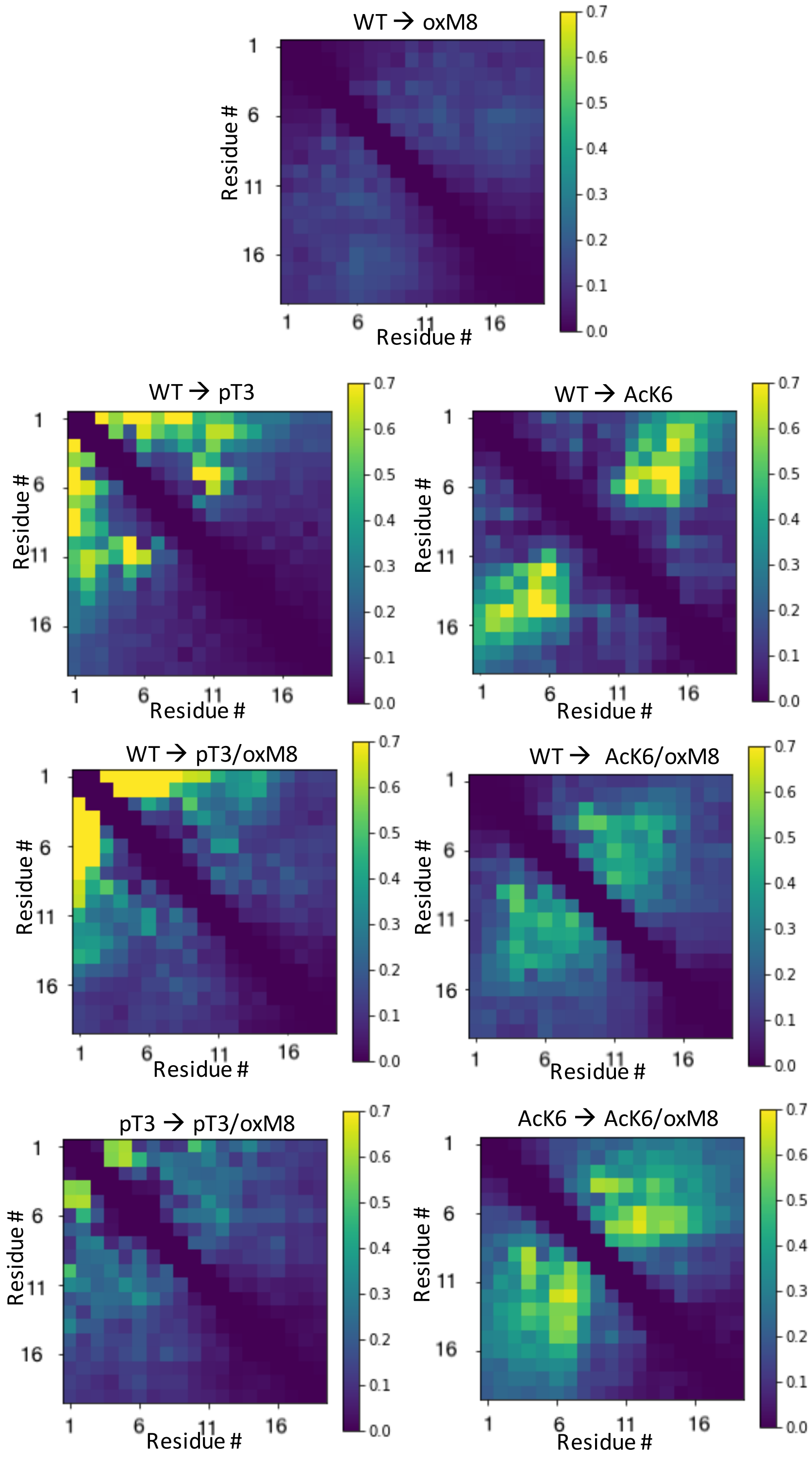

Figure S6. The Kullback–Leibler divergence of pairwise distances of Cα. For each MD of Nt19, the distances between all pairs of Cα in Nt19 were calculated. The differences in residue-residue contact were quantified using the Kullback-Leibler divergence from its reference histogram of distances.
